# 

**Published:** 1892-09-17

**Authors:** 


					.PRICE TWOPENCE.
1312, Vol. XII.?Sept. 17th, 1892.
^tered at the General Post Office ai a Newspaper for
btnamiasion in the United Kingdom and Abroad.]
f H>
WITH EXTRA NURSING SUPPLEMENT.
Per Annum, 8s. 6d., post free.
Monthly Farts. 6d.; post fiee, 8d.
Ditto per Annum, 8s., post free.
No. 312. Vol. XII.] Saturday,
September 17th, 1892.
TTwopenoe.

				

